# The Effects of Intra-Aortic Balloon Pumps on Mortality in Patients Undergoing High-Risk Coronary Revascularization: A Meta-Analysis of Randomized Controlled Trials of Coronary Artery Bypass Grafting and Stenting Era

**DOI:** 10.1371/journal.pone.0147291

**Published:** 2016-01-19

**Authors:** You-Dong Wan, Tong-Wen Sun, Quan-Cheng Kan, Fang-Xia Guan, Zi-Qi Liu, Shu-Guang Zhang

**Affiliations:** 1 Department of Integrated ICU, the First Affiliated Hospital of Zhengzhou University, Zhengzhou, PR China; 2 Pharmaceutical Department, the First Affiliated Hospital of Zhengzhou University, Zhengzhou, PR China; 3 Academy of Medical Science, Henan Province, Zhengzhou, PR China; San Raffaele Scientific Institute, ITALY

## Abstract

**Background:**

Intra-aortic balloon pumps (IABP) have generally been used for patients undergoing high-risk mechanical coronary revascularization. However, there is still insufficient evidence to determine whether they can improve outcomes in reperfusion therapy patients, mainly by percutaneous coronary intervention (PCI) with stenting or coronary artery bypass graft (CABG). This study was designed to determine the difference between high-risk mechanical coronary revascularization with and without IABPs on mortality, by performing a meta-analysis on randomized controlled trials of the current era.

**Methods:**

Pubmed and Embase databases were searched from inception to May 2015. Unpublished data were obtained from the investigators. Randomized clinical trials of IABP and non-IABP in high-risk coronary revascularization procedures (PCI or CABG) were included. In the case of PCI procedures, stents should be used in more than 80% of patients. Numbers of events at the short-term and long-term follow-up were extracted.

**Results:**

A total of 12 randomized trials enrolling 2155 patients were included. IABPs did not significantly decrease short-term mortality (relative risk (RR) 0.66; 95% CI, 0.42–1.01), or long-term mortality (RR 0.79; 95% CI, 0.47–1.35), with low heterogeneity across the studies. The findings remained stable in patients with acute myocardial infarction with or without cardiogenic shock. But in high-risk CABG patients, IABP was associated with reduced mortality (71 events in 846 patients; RR 0.40; 95%CI 0.25–0.67).

**Conclusion:**

In patients undergoing high-risk coronary revascularization, IABP did not significantly decrease mortality. But high-risk CABG patients may be benefit from IABP. Rigorous criteria should be applied to the use of IABPs.

## Introduction

Nowadays, coronary artery bypass graft (CABG) and percutaneous coronary intervention (PCI) with stents are the most common techniques of coronary revascularization. Although tremendous advances have been made in stent types, adjunctive pharmacotherapy, and surgical techniques, mortality rates are still high in high-risk patients [1].

As a circulatory-assist device, the intra-aortic balloon pump (IABP) has been shown to improve clinical outcomes and decrease the mortality rate in high-risk patients undergoing coronary revascularization [2]. However, current guidelines recommend using it only on patients with complications after st-segment elevation myocardial infarction[1]. In North America, China, and elsewhere, IABPs have been used in conjunction with medical therapy, to treat high-risk patients undergoing PCI or CABG[3], because of their potential benefits. During reperfusion treatment, adjunctive use of IABPs can significantly increase diastolic and mean blood pressure in the aorta and coronary artery and decrease systolic pressure, thereby unloading the heart. Additionally, it can improve myocardial perfusion at the tissue level, and reduce the extent of no-reflow caused by micro vascular obstruction[4]. Although animal trials have shown numerous advantages in the use of IABPs[4], the results from clinical trials are conflicting. While some trials[5] found that patients with acute myocardial infarction (AMI), complicated by cardiogenic shock, gained substantial benefits from IABPs, other trials were in disagreement[6]. Furthermore, a recent meta-analysis showed that IABPs significantly increase mortality in patients receiving PCI treatment[7], and these findings were confirmed in other similar studies[8–9].

For emergency high-risk patients, IABP has always been inserted before coronary revascularization. So it could be really important to decide whether or not to use an IABP before deciding using PCI or CABG. Therefore, we conducted this meta-analysis using rigorous inclusion criteria, and regardless of the type of revascularization, to evaluate the risks of IABPs on mortality in patients undergoing high-risk coronary revascularization in our current era of high arterial graft and stent use.

## Materials and Methods

### Literature Search

A systematic search of all published studies in Pubmed and Embase, from inception to May 2015, was independently performed by two members of our team (YD.W and TW.S). Search terms included: percutaneous coronary intervention, stent, CABG, coronary bypass, CAD, IABP, and intra-aortic balloon counterpulsation. We also reviewed reference lists and recent reviews to identify other potentially eligible studies that had not been identified in our initial search. Details of the search strategy are summarized in S1 Table.

### Study Selection

Studies were required to be RCTs of revascularization procedures, as well as IABP vs. revascularization procedures alone, in high-risk CAD patients with the individual outcomes of death. In addition, to reflect contemporary interventions and medical practice, inclusion criteria also required the revascularization procedures to be PCI or CABG, and stent implantation in at least 80% of PCI procedures. Finally, all high-risk factors had to be documented for all patients prior to randomization. We excluded reperfusion therapy studies with thrombolytic therapy or drug therapy.

### Data Extraction

Two members of our team (YD.W and ZQ.L) used a standardized data collection form to extract the following information from each included study: source, study design, number of IABP group cases, number of control group cases, population characteristics, definition of IABP and control group, IABP procedure and characteristics of the enrolled patients (age, gender, body-mass index, smoker, hypertension, diabetes, left ventricular ejection fraction (LVEF), prior MI, and stent placed). The supplementary files were also examined for data extraction. When necessary, we contacted authors of the included studies to obtain additional information. The quality of eligible RCTs was assessed using the Cochrane Collaboration's tool for assessing the risk of bias for RCTs [10].

### Statistical Analysis

Relative risk (RR) was used as a common measure of the association between IABP and mortality from PCI or CABG, across studies. Heterogeneity across trials was assessed using a standard chi-squared test, with significance being set at *P* < 0.10. Heterogeneity was also assessed by means of the *I*^*2*^ statistic, with significance being set at *I*^*2*^ > 50%. The random-effects model was used for statistical analyses due to the wide range of clinical and methodological variability across the trials. We further conducted subgroup analyses to explore possible explanations for heterogeneity. Sensitivity analyses were performed using the one-study-out method, and applying various exclusion criteria to test the robustness of the pooled estimate. Publication biases were evaluated using Funnel plots and Egger’s tests[11]. Statistical analyses were performed using STATA 12.0 (Stata Corp). A *P-*value <0.05 was considered to be statistically significant.

## Results

### Search Results

**Fig 1:**
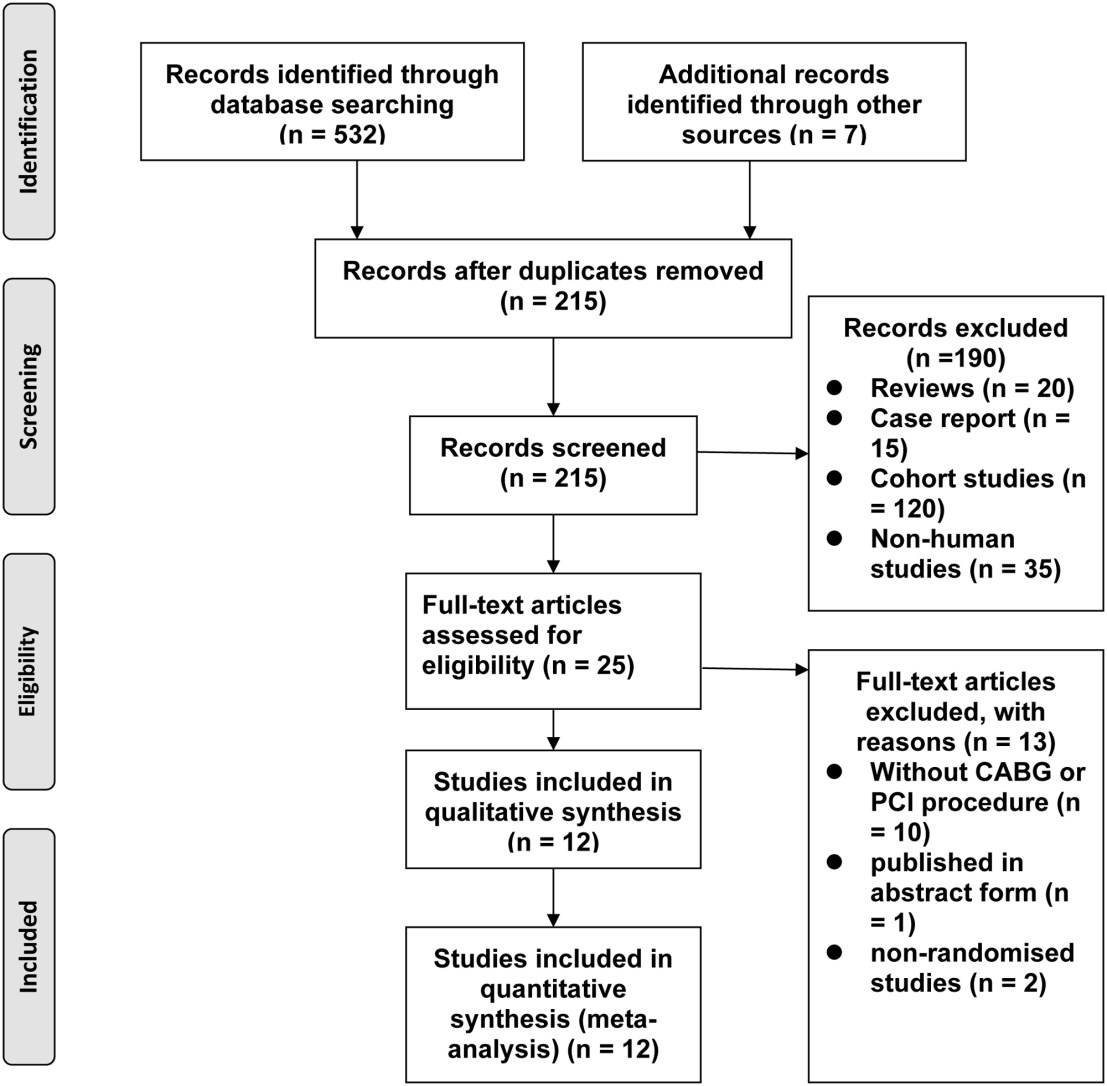
Flow diagram for selection of articles.

We performed this meta-analysis in accordance with the PRISMA statement[12] for reporting systematic reviews and meta-analyses. The electronic search yielded 215 citations, which were screened by reviewing the title or abstract. Of these, 25 publications were reviewed in full and 12 trials [13–26] consisting of 2155 patients (1067 in the IABP group and 1088 in the control group) were included in the meta-analysis (Fig 1). A study by Gu et al.[27] was excluded because the corresponding author confirmed that stent implantation rate data for the PCI group were missing. In addition, five papers[21–25] were published by the same author. However, it was confirmed by the author[21–25] in two previous meta-analyses[28, 29] that these five publications are on five different groups of patients, and it was not duplicate publications.

### Study and Patient Characteristics

The characteristics of the trials included in this study are listed in Table 1. Four trials[13–16,19] described PCI with stenting, seven trials[20–26] described CABG with arterial grafting, and one trial[17–18] described both PCI and CABG. In all trials, the IABP was inserted via the femoral artery. Three large international multicenter RCTs were included[14–18], while the other studies were all single center RCTs. Short-term mortality was often defined as in-hospital mortality, whereas long-term mortality meant death after 6 months or more. The BCIS-1 study looked at both 6-month[16] and 51-month[15] mortality; for the sake of consistency, we only included data on 6-month mortality[16]. The definition of “high-risk” was different across the studies. It mainly referred to AMI with or without cardiogenic shock, left ventricular dysfunction with low LVEF, left coronary main stem stenosis, and reoperation. Baseline patient characteristics are shown in Table 2. Because of a lack of baseline data in the studies by Christenson et al.[21–25], we did not include the patient characteristics from these studies. Baseline data in Table 2 showed that patient characteristics were similar in the IABP and control groups, but most patients had low LVEF (83% of patients with LVEF <40%). Because some IABP devices were not reliable and ethically suitable, double-blind designs were not selected for all trials. The quality of RCTs assessed using the Cochrane Collaboration's tool was shown in S2 Table.

**Table 1:** Characteristics of Included Trials.

Source	Designs	Period(year)	No. Randomized total (IABP/Control)	Patient characteristics	IABP group	Control group	IABP procedure	Definition of mortality	
								short-term mortality	long-term mortality
**IABP-SHOCK [13]**	RCT,singlecenter	2003–2004	40 (19/21)	Patients treated with primary PCI for cardiogenic shock secondary to acute myocardial infarction, who required inotropic and/or vasopressor support despite appropriate volume filling.	PCI + IABP	PCI	A 40 mL IABP balloon was inserted via the femoral artery using an 8-French sheath. Aortic counterpulsation was continued for a minimum of 48 hours	In-hospital mortality	NR
**CRISP-AMI[14]**	RCT, multicenter	2009–2011	337 (161/176)	Adult patients, six hours within the onset of chest pain, with a planned primary PCI for acute anterior st-segment elevation myocardial infarction with significant myocardium at risk, excluding cardiogenic shock	PCI + IABP	PCI	The intra-aortic balloon was inserted and pumped prior to PCI, balloon counterpulsation was recommended for at least 12 hours, for a maximum of 24 hours after PCI	30 day mortality	6 month mortality
**BCIS-1[15,16]**	RCT, multicenter	2005–2009	301 (151/150)	Patients with LVEF <30% with extensive myocardium at risk, excluding cardiogenic shock	PCI + IABP	PCI	Sheathed or unsheathed percutaneous insertion used prior to PCI, via the left or right femoral artery. The IABP remained *in situ* for at least four hours after PCI and was removed within 24 hours	In-hospital mortality	6 month mortality
**IABP-SHOCK II[17,18]**	RCT, multicenter	2009–2012	600 (301/299)	Patients with acute myocardial infarction (with or without ST-segment elevation) complicated by cardiogenic shock	PCI/CABG+ IABP	PCI/CABG	Intra-aortic balloon pumping insertion is performed via the femoral artery, with or without sheath insertion, before or directly after PCI	30 day mortality	1 year mortality
**Vijayalakshmi[19]**	RCT,singlecenter	NR	33 (17/16)	Patients undergoing emergency or urgent angiography (ST-elevation and non-ST-elevation myocardial infarction) with a view to angioplasty, excluding cardiogenic shock	PCI + IABP	PCI	The IABP catheter was inserted percutaneously via the femoral artery. Following insertion of the device, the IABP was used on a 1:1 ratio with full augmentation after PCI. The IABP was left *in situ* for up to 48 hours where possible	In-hospital mortality	NR
**SCORE[20]**	RCT,singlecenter	2009–2012	110 (55/55)	Adult patients scheduled for surgical myocardial revascularization with or without associated procedures, having a preoperative LVEF <35%, and stable hemodynamic conditions	CABG + IABP	CABG	Patients in the IABP group received a percutaneous femoral artery fiber-optic IABP after induction of the anesthesia and before skin incision. Balloon inflation was activated immediately after the positioning, interrupted during cardiopulmonary bypass, and restored after completion of the procedure to assist the weaning from the cardiopulmonary bypass	In-hospital mortality	NR
**Christenson[21]**	RCT,singlecenter	1997–1998	60 (30/30)	Patients who had CABG, presenting with two or more of the following criteria: LVEF ≤30%, unstable angina, reoperation, or left main stenosis >70%	CABG + IABP	CABG	40-ml IABP catheters were used and placed percutaneously. IABP therapy started two hours, 12 hours, or 24 hours, by random assignment, before the operation	In-hospital mortality	NR
**Christenson[22]**	RCT,singlecenter	1994–1996	52 (32/20)	Patients who had CABG, presenting with two or more of the following criteria: preoperative LVEF ≤40%, left main coronary artery stenosis ≥70%, reoperation and/or unstable angina despite medical treatment	CABG + IABP	CABG	IABPs were inserted via a percutaneous route (femoral artery), preoperatively, on average 1.5h, 24 h prior to the start of the cardiopulmonary bypass.	In-hospital mortality	NR
**Christenson[23]**	RCT,singlecenter	NR	30 (15/15)	Patients who had off-pump CABG, presenting with two or more of the following criteria: LVEF ≤ 30%, re-operative CABG, preoperative unstable angina, left coronary main stem stenosis >70%, recent myocardial infarction, less than 10 days prior to surgery	CABG + IABP	CABG	The IABP catheter was placed using a percutaneous insertion technique via a femoral artery. Preoperative insertion was normally performed	In-hospital mortality	NR
**Christenson[24]**	RCT,singlecenter	1994–1996	48(24/24)	Patients undergoing redo CABG, with at least two of the following additional criteria: LVEF ≤40%, unstable angina, left main stem stenosis ≥ 70%, or a combination of all criteria.	CABG + IABP	CABG	IABP catheters were inserted via a percutaneous route (femoral artery) on average two hours before cardiopulmonary bypass	In-hospital mortality	NR
**Christenson[25]**	RCT,singlecenter	1994–1996	33 (19/14)	All patients had a preoperative LVEF ≤ 40%, 3-vessel disease, established hypertension, and left ventricular hypertrophy.	CABG + IABP	CABG	IABP catheters were inserted via a percutaneous route (femoral artery) on average two hours before cardiopulmonary bypass	In-hospital mortality	NR
**Wilczynski[26]**	RCT,singlecenter	2004–2008	502 (243/259)	Patients who met at least two of the following criteria: LVEF < 35%, unstable angina, left main stem stenosis of at least 80% or redo CABG, and simultaneously had a EuroSCORE of plus 6.	CABG + IABP	CABG	Preoperative insertion of IABP was performed in the operating room, one hour prior to surgery.	In-hospital mortality	NR

RCT, randomized controlled trial; PCI, percutaneous coronary intervention; CABG, coronary artery bypass grafting; IABP, intra-aortic balloon pump; LVEF: left ventricular ejection fraction; NR, not reported.

**Table 2:** Characteristics of Enrolled Patients.

Source	Age(mean, y)		Male(%)		Body-mass index (mean, μg/m^2^)		Smoker(%)		Hypertension (%)		Diabetes (%)		LVEF(%)		Prior MI (%)		Stent placed(%)	
	IABP	Control	IABP	Control	IABP	Control	IABP	Control	IABP	Control	IABP	Control	IABP	Control	IABP	Control	IABP	Control
**IABP-SHOCK [13]**	62	66	74	81	28	28	42	33	42	48	53	48	37	38	21	24	84	86
**CRISP-AMI[14]**	56	58	82	82	NR	NR	33	31	24	34	17	21	46	48	0	0	97	94
**BCIS-11[15,16]**	71	71	81	78	28	27	21	20	63	61	37	33	24	24	75	73	94	93
**IABP-SHOCK II[17,18]**	70	69	67	71	28	27	33	36	72	67	35	30	35	35	24	20	90	89
**Vijayalakshmi[19]**	NR	NR	82	88	NR	NR	35	50	35	38	18	25	NR	NR	12	25	100	100
**SCORE[20]**	67	66	87	87	26	25	NR	NR	NR	NR	24	15	28	30	55	20	NA	NA
**Wilczynski[26]**	NR	NR	NR	NR	NR	NR	NR	NR	45	51	22	23	37	36	44	48	NA	NA

LVEF: left ventricular ejection fraction; NR, not reported; NA, not available.

### Quantitative Data Synthesis

**Fig 2:**
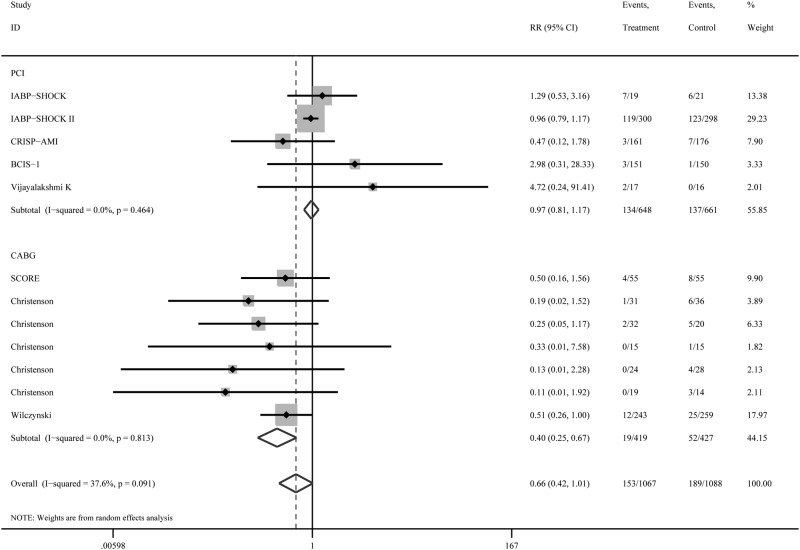
Subgroup results by type of revascularization for short-term mortality according to treatment arm.

**Fig 3:**
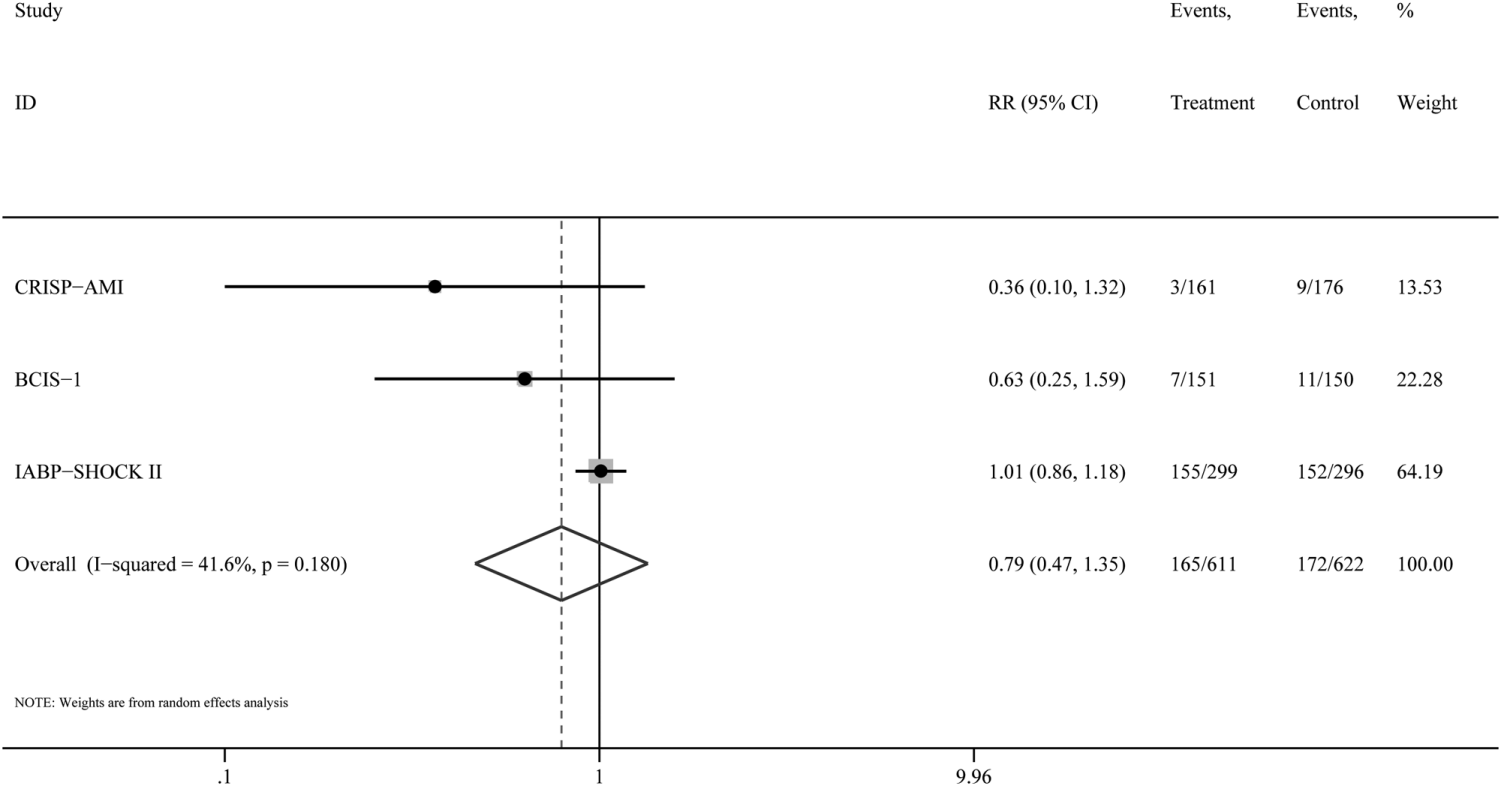
Long-term mortality according to treatment arm.

The pooled results from the random-effects model combining the RRs for short-term mortality are shown in Fig 2. Of the 342 deaths among 2155 randomized patients with coronary revascularization, 153 deaths occurred in 1067 patients (14.3%) randomized to the IABP group, whereas 189 deaths occurred in 1088 patients (17.4%) randomized to the control group. IABP was not associated with a significant reduction in short-term mortality (RR 0.66; 95% CI, 0.42–1.01), with low heterogeneity among the studies (*I*^*2*^ = 37.6%, P = 0.091). Similarly, in three studies[16–20] on 1233 patients, no long-term mortality advantage was observed in the IABP group (RR 0.79; 95% CI, 0.47–1.35), with low heterogeneity (*I*^*2*^ = 41.6%, P = 0.180) (Fig 3).

### Subgroup Analyses

**Fig 4:**
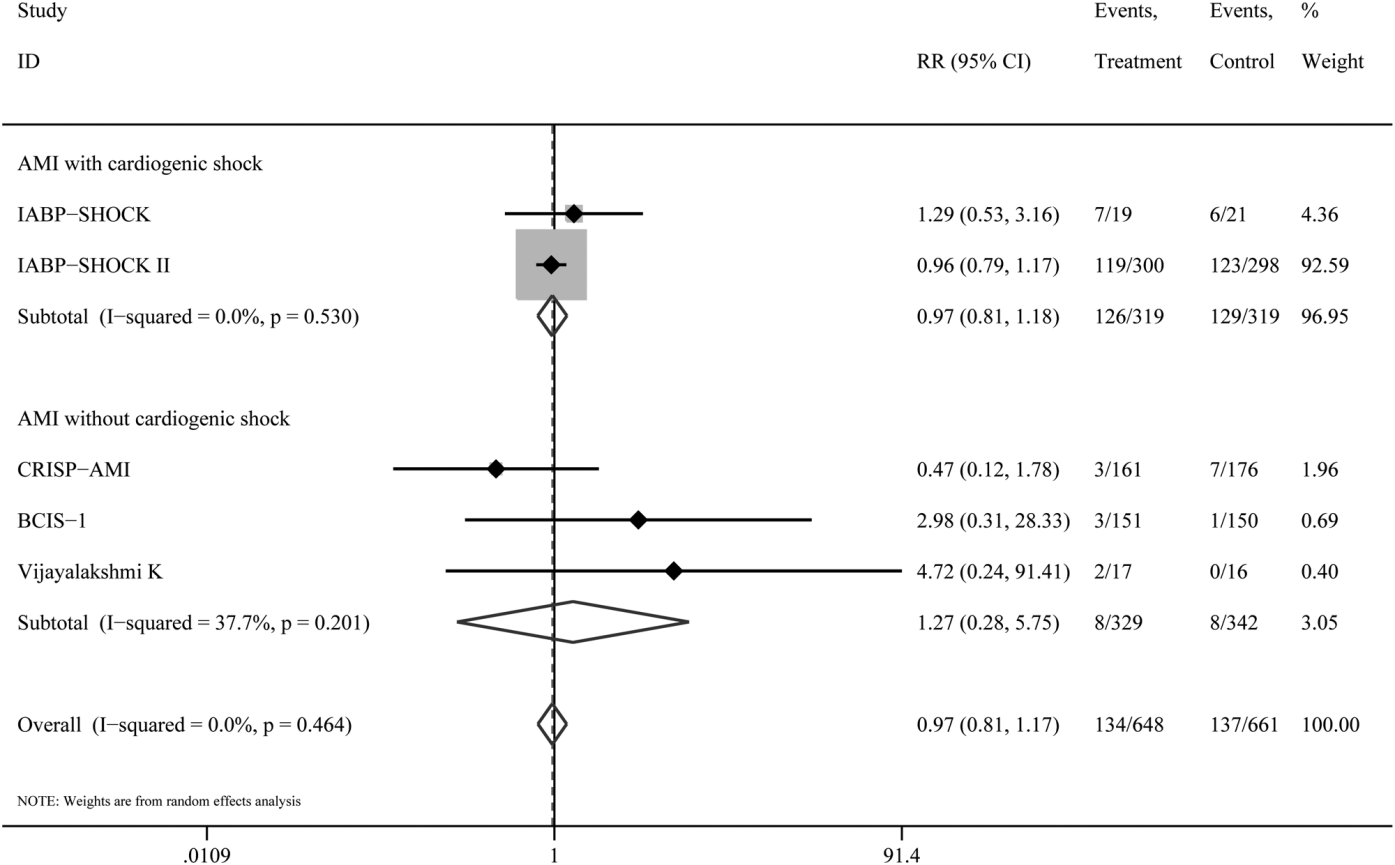
Subgroup results by type of disease for short-term mortality according to treatment arm.

We performed a subgroup analysis for short-term mortality according to the type of disease (AMI with or without cardiogenic shock) and the type of revascularization (PCI or CABG). The mortality was not significantly decreased in patients with IABP experiencing AMI with cardiogenic shock (RR 0.97; 95% CI, 0.81–1.18), AMI without cardiogenic shock (RR 1.27; 95% CI, 0.28–5.70)(Fig 4). Similarly, The mortality in patients with IABP did not significantly decreased following revascularization by PCI (RR 0.97; 95% CI, 0.81–1.17), but not CABG (RR 0.40; 95% CI, 0.25–0.67)(Fig 2). There was significant subgroup differences between PCI and CABG group (P = 0.001).

### Sensitivity Analyses and Publication Bias

**Fig 5:**
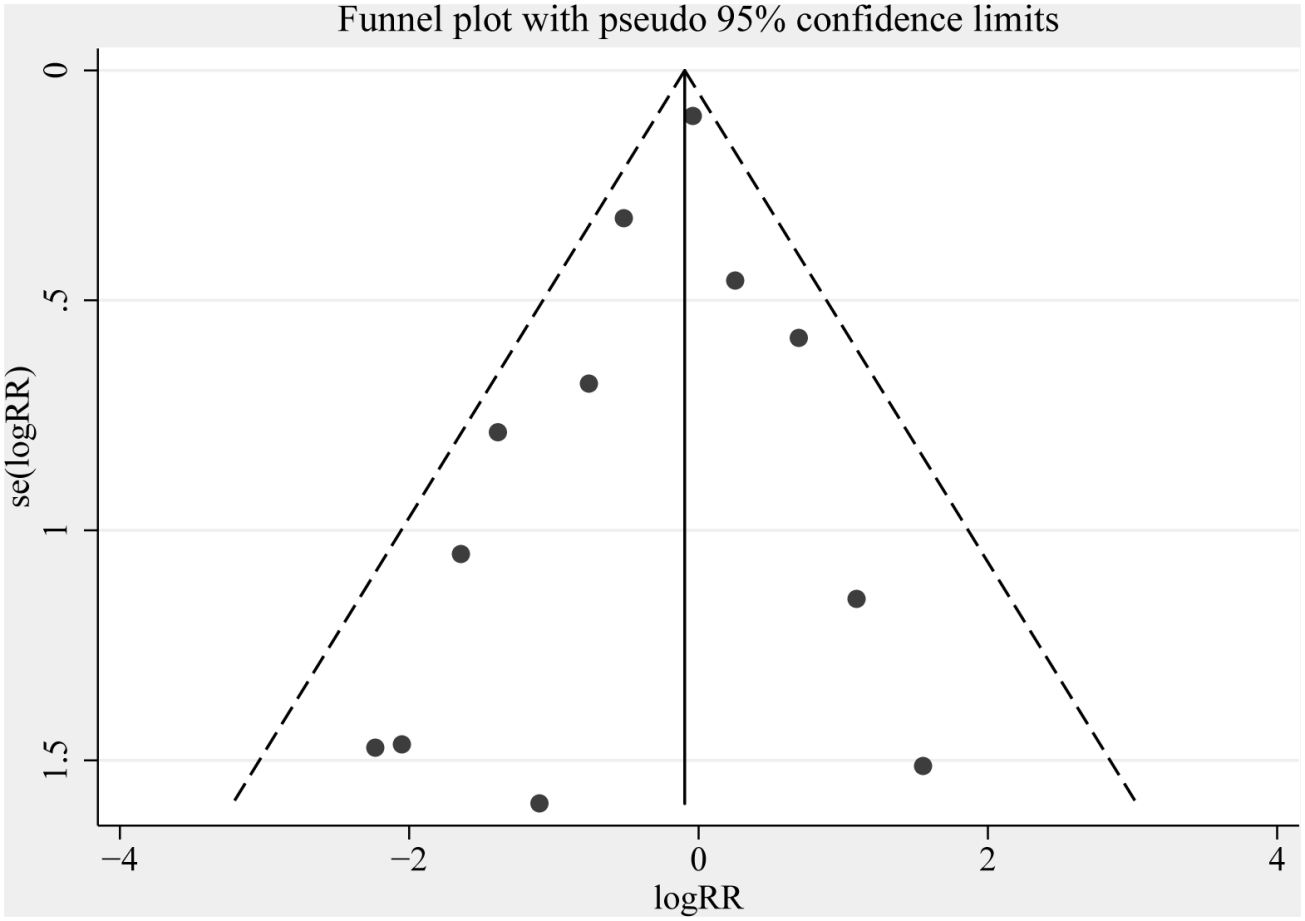
Funnel plots examining publication bias for short-term mortality.

Our sensitivity analyses indicate that the various exclusion criteria that were employed (single centers, small sample groups, and studies by Christenson et al.) did not make a significant difference, and the findings of our meta-analysis remained stable despite the exclusion of any single study (S3 Table). No evidence of publication bias was detected when this issue was examined by Egger’s tests and funnel plots. The test statistic for the Egger approach was non-significant for reporting on short-term (*P* = 0.13) and long-term mortality (*P* = 0.25), and the funnel plot was symmetric (Fig 5).

## Discussion

Our meta-analysis on 12 RCTs including 2155 patients suggests that IABPs did not significantly decrease short-term mortality and long-term mortality. In addition, The mortality of IABP group did not significantly decreased in MI patients with or without cardiogenic shock. But a survival advantage was found in high risk CABG patiens.

IABPs have been widely used since the 1960s, and are still a common technique. However, its effects on mortality are still controversial. In a large National Registry of 23,180 patients with cardiogenic shock, a significant reduction in mortality rates was observed[30]. However, the TACTICS trial[31] randomized 57 patients and found that early IABP use was not associated with definitive survival benefits in patients with MI and hemodynamic compromise. A meta-analysis,[32] including seven randomized trials and 1009 patients with ST-elevation myocardial infarction, showed that IABP did not yield 30-day survival benefits nor did it improve LVEF. Another Bayesian approach network meta-analysis [33] including 9 randomized trials comparing IABP and medical therapy, also found no survival advantage of IABP. Recently, Su et al [34] including 17 studies with 3226 AMI patients, the results still did not favor the use of IABP. The current 2013 ACCF/AHA guidelines recommend (class IIa) the use of IABP in patients with cardiogenic shock after st-segment elevation myocardial infarction (Level of Evidence: B). However, in most of these studies, the method for reperfusion therapy was thrombolytic therapy, while arterial grafting and stenting, which are more commonly used nowadays, may not be suitable for current clinical practice. On the other hand, the use of IABPs is only recommended in patients with MI or cardiogenic shock, and there is very little evidence of probable benefits for any form of high-risk revascularization. There is an urgent need to determine whether IABPs should be more widely used. Our meta-analysis indicates that IABPs do not decrease short-term or long-term mortality, so they should be used with caution.

Another interesting finding supported the use of prophylactic IABPs in high-risk CABG patients to reduce mortality, and this was consistent with previous meta-analyses[28,29,35]. One recent meta-analysis[28] analyzed six RCTs from the network database and one RCT from a conference paper with a total of 345 patients. The study showed that IABPs could reduce hospital mortality; however, out of these seven RCTs, five were published by the same author, so there may have been a bias in the results[21–25]. In our meta-analysis, we excluded one unsuitable trial[36], focusing on the timing of IABP support; we also excluded a low-quality conference paper. We replaced these two studies with two large high-quality RCTs[21,26] on 612 additional patients. The findings of these two RCTs further supported our conclusions. A meta-analysis[37] involving ten RCTs on 2037 patients showed that IABP use in high-risk patients undergoing reperfusion therapy can improve long-term mortality. However, in this meta-analysis, “high-risk reperfusion therapy” means PCI (with or without stents) and thrombolysis therapy, but not CABG. In addition, the study period ranged from 1994 to 2012, when tremendous advances were being made on stents and adjunctive pharmacotherapy used with PCI, so the results may not be applicable at present. Another meta-analysis[38] included 9 RCTs with 1171 patients undergoing high-risk cardiac surgery, the results were consistent with our subgroup analysis results. In our meta-analysis, the data from three large international multicenter RCTs[14–18] do not support the use of IABPs to reduce mortality.

Our study showed that survival advantage was found in high risk CABG patients, but not PCI patients. This conflicting result may be explained by the following two reasons. First, The advancement in PCI technology and cardiac medications are one possible explanations. The PCI technology, stent material, and treatment concept advanced greatly, which result to decreased mortality in AMI patients. The loss of benefit of IABP could be remedied by the use of effective cardiac medications. Therefore, it might be hypothesized that the IABP might not show any beneficial effect due to improved care [38]. Another reason was that patients undergoing PCI do not suffer from anaesthesia or institution of extracorporeal circulation. The benefit of IABP mainly included adding the cardiac support and reducing the incidence of haemodynamic complications. Therefore, the IABP could improve myocardial perfusion during the procedural haemodynamic disturbances of CABG.

Our findings may have several significant advantages. First, to reflect current clinical practice, the inclusion criteria required all revascularization procedures to be PCI or CABG, with stent implantation in at least 80% of PCI procedures. As a result, all studies about PCI with stenting were published in the last 5 years. Given that the first published RCTs about IABP in PCI patients were conducted as early as 1989 years by Ohman EM et al[39], when the technique of reperfusion therapy has developed rapidly over the recent 20 years. Including these trials in the meta-analysis may result in unadjusted heterogeneity, and the results cannot be completely applied to patients in this era. Therefore, we pre-stated rigorous inclusion criteria to adapt to current clinical practice. Second, all included studies were RCTs, which could avoid some potential biases. Observational studies are always at risk of unmeasured confounding variables, which are inherent to the study design and cannot be avoided. Among these biases, the “healthy user” bias[40], which states that IABP users tend to have less severe comorbidity, a better functional status, and are healthier than non-users, is a major source of potentially confounding variables. If we take as an example a large national registry of patients with cardiogenic shock, treated by IABP [30], the patients in the IABP group were younger, more often male, with a significantly lower history of diseases (diabetes, congestive heart failure, prior AMI, prior CABG, and so on). This result could be explained by the fact that young males are more likely to try the IABP procedure because they tend to be healthier, with a better functional status, and a more adventurous spirit. In a multivariate model of this national registry, an increase in age, incidence of diabetes, and history of diseases, showed an increase in mortality rates. Although using a propensity score should reduce the possible bias, the results are still likely to overestimate the true magnitude of the effect of IABPs. Such biases are not present when analyzing data from RCTs only. Based on this, our meta-analysis, which shows that IABPs have no therapeutic effects on patients undergoing high-risk coronary revascularization, is probably a truly representative report.

Several limitations of this meta-analysis merit consideration. First, the time when the IABP was inserted and removed (before or after PCI and CABG), and the type of IABP device employed, were not strictly described in all trials. Second, there were no long-term mortality data available for some RCTs[13,19–26]. Third, we were unable to assess the impact of IABPs on other clinically meaningful end points, such as the risk of moderate and major bleeding events and complications from the IABP, because reporting across studies was sparse and inconsistent. According to the IABP-SHOCK II trial[18], the bleeding rate (moderate and severe bleeding) for patients who were in hospital with MI complicated by cardiogenic shock was about 20%, which was quite high. Further studies should focus on this and the complications arising from IABP.

## Conclusions

For patients undergoing high-risk coronary revascularization with PCI, the routine use of IABPs does not reduce the risk of death compared to no IABPs. But high-risk CABG patients may be benifit from IABP. These findings underline the importance of rigorous criteria in using IABPs to avoid possible complications.

## Supporting Information

S1 Table Search strategy. The electronic search strategy without language limitation.
(DOCX)

S2 Table Risk of bias of included studies. The risk of bias assessed with Cochrane Collaboration's tool.
(DOCX)

S3 Table Sensitivity analyses for the outcome of short-term mortality. (DOCX)

S4 Table PRISMA Checklist. Checklist of preferred reporting items for systematic reviews and meta-analyses.
(DOC)
