# Supplementary material for: The Effects of Intra-Aortic Balloon Pumps on Mortality in Patients Undergoing High-Risk Coronary Revascularization: A Meta-Analysis of Randomized Controlled Trials of Coronary Artery Bypass Grafting and Stenting Era
Source: PLoS One. 2016 Jan 19;11(1):e0147291. doi: 10.1371/journal.pone.0147291 (PMC4718717; doi:10.1371/journal.pone.0147291)
Supplement: S1 Table — The electronic search strategy without language limitation. (DOCX) [file pone.0147291.s001.docx]

- Search strategy (pubmed as instance)

| **Search** | **Query** | **Items found** |
| --- | --- | --- |
| **#4** | ((#1) AND #2) AND #3 | 166 |
| **#3** | ((randomized controlled trial[pt] OR controlled clinical trial[pt] OR randomized[tiab] OR placebo[tiab] OR clinical trials as topic[mesh:noexp] OR randomly[tiab] OR trial[ti]) NOT (animals[mh] NOT humans[mh])) | 915927 |
| **#2** | ((((coronary bypass) OR percutaneous coronary intervention) OR stent) OR CABG) OR Coronary Artery Bypass Grafting | 163375 |
| **#1** | ((intra-aortic balloon counterpulsation) OR intra-aortic ballon pump) OR IABP | 4366 |

- **The following related reviews is also checked**

1. Sjauw KD, Engstrom AE, Vis MM, van der Schaaf RJ, Baan JJ, et al. A systematic review and meta-analysis of intra-aortic balloon pump therapy in ST-elevation myocardial infarction: should we change the guidelines? Eur Heart J. 2009; 30(4):459-468.
2. Bahekar A, Singh M, Singh S, Bhuriya R, Ahmad K, Khosla S, et al. Cardiovascular outcomes using intra-aortic balloon pump in high-risk acute myocardial infarction with or without cardiogenic shock: a meta-analysis. J Cardiovasc Pharmacol Ther. 2012; 17(1):44-56.
3. Altayyar S, Rochwerg B, Alnasser S, Al-Omari A, Baw B, Fox-Robichaud A, et al. Intra-aortic balloon pump in patients with cardiogenic shock complicating myocardial infarction: a systematic review and meta-analysis of randomized trials (protocol). Syst Rev. 2014; 3:24.
4. Su D, Yan B, Guo L, Peng L, Wang X, Zeng L, et al. Intra-aortic balloon pump may grant no benefit to improve the mortality of patients with acute myocardial infarction in short and long term: an updated meta-analysis. Medicine (Baltimore). 2015; 94(19):e876.
5. Ahmad Y, Sen S, Shun-Shin MJ, Ouyang J, Finegold JA, Al-Lamee RK, et al. Intra-aortic Balloon Pump Therapy for Acute Myocardial Infarction: A Meta-analysis. JAMA Intern Med. 2015; 175(6):931-939.
6. Romeo F, Acconcia MC, Sergi D, Romeo A, Gensini GF, Chiarotti F, et al. Lack of intra-aortic balloon pump effectiveness in high-risk percutaneous coronary interventions without cardiogenic shock: a comprehensive meta-analysis of randomised trials and observational studies. Int J Cardiol. 2013; 167(5):1783-1793.
7. Cheng JM, den Uil CA, Hoeks SE, van der Ent M, Jewbali LS, van Domburg RT, et al. Percutaneous left ventricular assist devices vs. intra-aortic balloon pump counterpulsation for treatment of cardiogenic shock: a meta-analysis of controlled trials. Eur Heart J. 2009; 30(17):2102-2108.
8. Dyub AM, Whitlock RP, Abouzahr LL, Cina CS. Preoperative intra-aortic balloon pump in patients undergoing coronary bypass surgery: a systematic review and meta-analysis. J Card Surg. 2008; 23(1):79-86.
9. Zangrillo A, Pappalardo F, Dossi R, Di Prima AL, Sassone ME, Greco T, et al. Preoperative intra-aortic balloon pump to reduce mortality in coronary artery bypass graft: a meta-analysis of randomized controlled trials. Crit Care. 2015; 19:10.
10. Sa MP, Ferraz PE, Escobar RR, Martins WN, Nunes EO, Vasconcelos FP, et al. Prophylactic intra-aortic balloon pump in high-risk patients undergoing coronary artery bypass surgery: a meta-analysis of randomized controlled trials. Coron Artery Dis. 2012; 23(7):480-486.
11. Chen S, Yin Y, Ling Z, Krucoff MW. Short and long term effect of adjunctive intra-aortic balloon pump use for patients undergoing high risk reperfusion therapy: a meta-analysis of 10 international randomised trials. Heart. 2014; 100(4):303-310.
12. Romeo F, Acconcia MC, Sergi D, Romeo A, Muscoli S, Valente S, et al. The outcome of intra-aortic balloon pump support in acute myocardial infarction complicated by cardiogenic shock according to the type of revascularization: a comprehensive meta-analysis. Am Heart J. 2013; 165(5):679-692.
