# Supplementary material for: The Effects of Intra-Aortic Balloon Pumps on Mortality in Patients Undergoing High-Risk Coronary Revascularization: A Meta-Analysis of Randomized Controlled Trials of Coronary Artery Bypass Grafting and Stenting Era
Source: PLoS One. 2016 Jan 19;11(1):e0147291. doi: 10.1371/journal.pone.0147291 (PMC4718717; doi:10.1371/journal.pone.0147291)
Supplement: S2 Table — The risk of bias assessed with Cochrane Collaboration's tool. (DOCX) [file pone.0147291.s002.docx]

**S2 Table. T****he risk of bias assessed with Cochrane Collaboration's tool**

| **Source** | **Random sequence generation**  **(selection bias)** | **Allocation concealment**  **(selection bias)** | **Blinding of participants and personnel  (performance bias)** | **Blinding of outcome assessment (detection bias)** | **Incomplete outcome data (attrition bias)** | **Selective reporting (reporting bias)** | **Other bias** |
| --- | --- | --- | --- | --- | --- | --- | --- |
| **IABP-SHOCK [13]** | **+** | **—** | **—** | **—** | **+** | **+** | **?** |
| **CRISP-AMI[14]** | **+** | **?** | **—** | **+** | **+** | **+** | **?** |
| **BCIS-1[15, 16]** | **_+_** | **—** | **—** | **+** | **+** | **+** | **?** |
| **IABP-SHOCK II[17, 18]** | **?** | **—** | **—** | **？** | **+** | **+** | **?** |
| **Vijayalakshmi[19]** | **+** | **?** | **?** | **+** | **+** | **+** | **?** |
| **SCORE[20]** | **+** | **?** | **—** | **+** | **+** | **+** | **?** |
| **Christenson[21]** | **?** | **?** | **—** | **?** | **+** | **+** | **?** |
| **Christenson[22]** | **?** | **?** | **—** | **?** | **+** | **+** | **?** |
| **Christenson[23]** | **?** | **?** | **—** | **?** | **+** | **+** | **?** |
| **Christenson[24]** | **?** | **?** | **—** | **?** | **+** | **+** | **?** |
| **Christenson[25]** | **?** | **?** | **—** | **?** | **+** | **+** | **?** |
| **Wilczynski[26]** | **+** | **—** | **—** | **+** | **+** | **+** | **?** |

The symbol(+) corresponds to low risk of bias, the symbol (?) corresponds to unclear risk of bias, and the symbol (-) corresponds to high risk of bias.
