# Supplementary material for: The Effects of Intra-Aortic Balloon Pumps on Mortality in Patients Undergoing High-Risk Coronary Revascularization: A Meta-Analysis of Randomized Controlled Trials of Coronary Artery Bypass Grafting and Stenting Era
Source: PLoS One. 2016 Jan 19;11(1):e0147291. doi: 10.1371/journal.pone.0147291 (PMC4718717; doi:10.1371/journal.pone.0147291)
Supplement: S3 Table — (DOCX) [file pone.0147291.s003.docx]

| **Characteristic** | **Number of studies** | **Results (RR, 95%CI)** |
| --- | --- | --- |
| **Multicenter studies** | 5 | 0.95(0.71,1.27) |
| **Sample size(number of patients>100)** | 5 | 0.90(0.61,1.34) |
| **Without studies from Christenson** | 9 | 0.96(0.70,1.31) |
| **Excluded Study in One-Study-Out Model** |  |  |
| **IABP-SHOCK** | 1 | 0.68(0.41,1.12) |
| **CRISP-AMI** | 1 | 0.78(0.49,1.25) |
| **BCIS-1** | 1 | 0.71(0.46,1.11) |
| **IABP-SHOCK II** | 1 | 0.67(0.37,1.20) |
| **Vijayalakshmi** | 1 | 0.72(0.46,1.13) |
| **SCORE** | 1 | 0.67(0.43,1.07) |
| **Christenson** | 1 | 0.80(0.52,1.23) |
| **Christenson** | 1 | 0.82(0.53,1.25) |
| **Christenson** | 1 | 0.76(0.48,1.20) |
| **Christenson** | 1 | 0.78(0.51,1.21) |
| **Christenson** | 1 | 0.79(0.51,1.21) |
| **Wilczynski** | 1 | 0.81(0.49,1.33) |

S3 Table. Sensitivity analyses for the outcome of short-term mortality.
